# B cell clonal expansion and convergent antibody responses to SARS-CoV-2

**DOI:** 10.21203/rs.3.rs-27220/v1

**Published:** 2020-05-06

**Authors:** Sandra C. A. Nielsen, Fan Yang, Ramona A. Hoh, Katherine J. L. Jackson, Katharina Roeltgen, Ji-Yeun Lee, Arjun Rustagi, Angela J. Rogers, Abigail E. Powell, Peter S. Kim, Taia T. Wang, Benjamin Pinsky, Catherine A. Blish, Scott D. Boyd

**Affiliations:** Department of Pathology, Stanford University; Department of Pathology, Stanford University; Department of Pathology, Stanford University; Garvan Institute of Medical Research; Department of Pathology, Stanford University; Department of Pathology, Stanford University; Department of Medicine, Division of Infectious Diseases and Geographic Medicine, Stanford University; Department of Medicine, Division of Pulmonary, Allergy and Critical Care Medicine, Stanford University; Stanford ChEM-H and Department of Biochemistry, Stanford University; Stanford ChEM-H and Department of Biochemistry, Stanford University; Department of Medicine, Division of Infectious Diseases and Geographic Medicine, Stanford University; Department of Pathology, Stanford University; Department of Medicine, Division of Infectious Diseases and Geographic Medicine, Stanford University; Department of Pathology, Stanford University

**Keywords:** SARS-CoV-2, B cells, antigen receptors

## Abstract

During virus infection B cells are critical for the production of antibodies and protective immunity. Establishment of a diverse antibody repertoire occurs by rearrangement of germline DNA at the immunoglobulin heavy and light chain loci to encode the membrane-bound form of antibodies, the B cell antigen receptor. Little is known about the B cells and antigen receptors stimulated by the novel human coronavirus SARS-CoV-2. Here we show that the human B cell compartment in patients with diagnostically confirmed SARS-CoV-2 and clinical COVID-19 is rapidly altered with the early recruitment of B cells expressing a limited subset of V genes, and extensive activation of IgG and IgA subclasses without significant somatic mutation. We detect expansion of B cell clones as well as convergent antibodies with highly similar sequences across SARS-CoV-2 patients, highlighting stereotyped naïve responses to this virus. A shared convergent B cell clonotype in SARS-CoV-2 infected patients was previously seen in patients with SARS. These findings offer molecular insights into shared features of human B cell responses to SARS-CoV-2 and other zoonotic spillover coronaviruses.

## Background

The novel human severe acute respiratory syndrome coronavirus 2 (SARS-CoV-2) is the etiological agent of the coronavirus disease 2019 (COVID-19)^1^,^2^ pandemic. Prior to the emergence of SARS-CoV-2, six human coronaviruses (hCoVs) were known; four seasonal hCoVs (hCoV-229E, -NL63, -HKU1, and -OC43)^3^ causing usually mild upper respiratory illness, and the two more recently discovered SARS-CoV^4^ and MERS-CoV^5^ viruses that arose from spillover events of virus from animals into humans. It is expected that humans are naïve to SARS-CoV-2 and will display a primary immune response to infection. Humoral immune responses will likely be critical for the development of protective immunity to SARS-CoV-2, but little is known about the B cells and antigen receptors stimulated by infection.

## Sars-cov-2 Infection Causes Global Changes In The Antibody Repertoire

**Figure 1:**
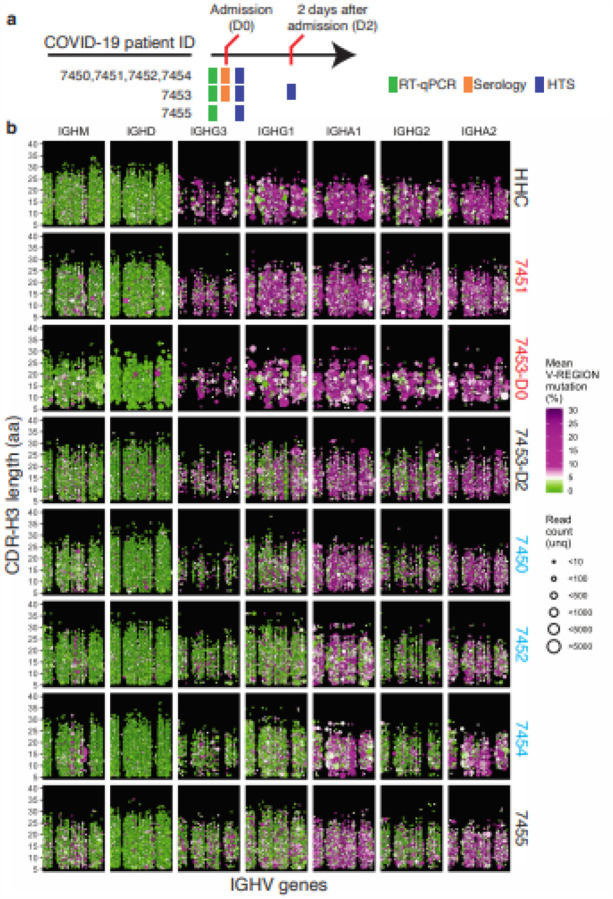
COVID-19 patient IGH repertoires show early and extensive class-switching to IgG and IgA subclasses without significant somatic mutation. **a**, Blood was collected from six COVID-19 patients at admission to hospital (D0) and for one patient again two days later (D2). Colored bars indicate assays and experiments performed on samples (RT-qPCR: green; Serology: orange; high-throughput sequencing (HTS): blue). **b**, Points indicate B cell clonal lineages, with the position denoting the clone’s isotype (panel column), human healthy control (HHC) or patient ID (panel row), IGHV gene (x-axis, with IGHV gene in the same order and position in panels, but not listed by name due to space constraints), and CDR-H3 length (y-axis within each panel). The point color indicates the mean IGHV SHM frequency for each clone and the size indicates the number of unique reads grouped into the clone. Points are jittered to decrease over-plotting of clones with same IGHV gene and CDR-H3 length. Patient label colors indicate seroconversion (blue), seronegative (red), and no serology performed (black). See Extended Data Fig. 2 for an overview of the IgG4 and IgE subclasses.

High-throughput DNA sequencing of B cell receptor (BCR) heavy chain genes defines clonal B cell lineages based on their unique receptor sequences, and captures the hallmarks of clonal evolution, such as somatic hypermutation (SHM) and class switch recombination, during the evolving humoral response^6^. To study the development of SARS-CoV-2-specific humoral responses, we collected peripheral blood samples from six patients admitted to Stanford Hospital with signs and symptoms of COVID-19. All patients were confirmed positive for SARS-CoV-2 RNA as determined by quantitative reverse transcription PCR (RT-qPCR). Serology of SARS- CoV-2 -specific IgA, IgG, and IgM antibodies showed early detection of these antibody (Fig. 1 a and Extended Data Fig. 1). Immunoglobulin heavy chain (IGH) repertoires were sequenced and compared to a healthy human control (HHC) dataset^7^ matched by age and number of B cell clones (Fig. 1 b, top panel). In healthy subjects at baseline, IgM and IgD sequences are primarily derived from naïve B cells with unmutated IGHV genes, and typically have small clone sizes, whereas isotype switched cells (IgA and IgG compartments) have elevated SHM with varying clone sizes. SARS-CoV-2 seroconverted patients (7450, 7452, and 7454), and patients 7453-D2 and 7455, in contrast, display a highly polyclonal burst of IgG-expressing clones in the blood with little to no SHM. Seronegative samples (7451 and 7453-D0) show IGH repertoires similar to uninfected controls, suggesting an earlier stage in the infection at the time of sample collection.

**Figure 2:**
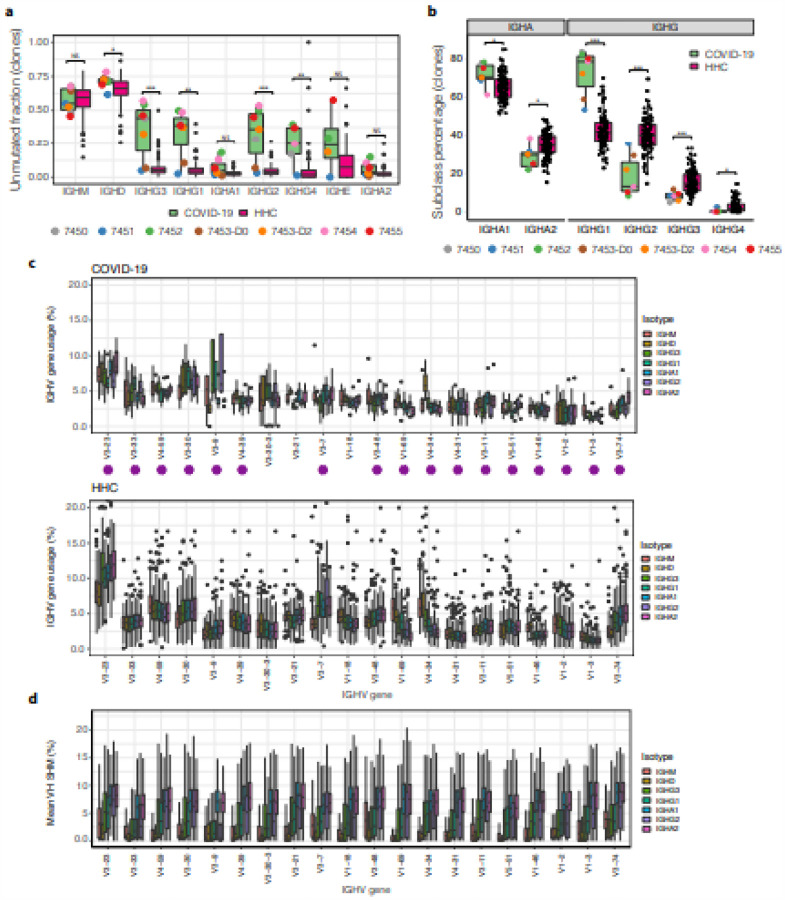
SARS-CoV-2 infection alters isotype mutation frequencies, subclass proportions, and usage of IGHV genes in the IGH repertoire. **a**, Fraction of unmutated (<1 % SHM) B cell lineages within each isotype subclass. Points are shown for all COVID-19 patients, but only outlier points are displayed for the 114 healthy human controls (HHCs). **b**, Isotype subclass usage within IgA and IgG. (**C**) IGHV gene usage is shown as the mean for clones within each COVID-19 patient (top) or HHC (bottom). IGHV order is based on the 20 most common IGHV genes in IgM in the COVID-19 patients and isotypes are plotted by their chromosomal ordering. The plot axes were chosen to show the box-whiskers on a readable scale; rare outlier points with extreme values are not shown but were included in all analyses. Asterisks represent IGHV genes that are used by reported SARS-CoV-2-specific IGH^10^. **d**, SHM frequency of combined clones from COVID-19, ordered by IGHV gene usage. p-values in (**a**) and (**b**) were calculated by two-sided Wilcoxon-Mann-Whitney tests: ***p-value < 0.001; **p-value < 0.01; *p-value ≤ 0.05; NS: p-value > 0.05.

As suggested by Fig. 1b, COVID-19 patients have a significantly increased fraction of unmutated and low mutation (<1 % SHM in IGHV gene) clonal lineages among the antigen-experienced, class switched IgG subclasses (IgG1: p-value = 0.00112; IgG2: p-value = 0.000667; IgG3: p-value = 0.000882; IgG4: p-value = 0.00667) (Fig. 2a). Among some seroconverted subjects (7452 and 7454), 50% or more of the IgG3, IgG1, and IgG2 compartments are in this low mutational state, compared to <1 % of lineages from corresponding HHC compartments (Fig. 2a). A similar influx of low mutation lineages into the IgG compartment has previously been observed in response to Ebola virus (EBOV) infection^8^. In contrast to EBOV, we observe that COVID-19 primary infection stimulates polyclonal B cell responses with both IgG and, in some patients, IgA subclasses, rather than predominantly IgG alone (Fig. 1 b). The SARS-CoV-2 response features a preponderance of IgG1-expression among IgG subclasses (Fig 2b), with median usage of IgG1 being almost double the proportion seen in HHC B cells (p-value = 2.065e-05). IgA1 is also significantly increased (p-value = 0.0123) relative to IgA2. This suggests an influx of recently recruited naTve B cells in the response. The virus-induced response is polyclonal with diverse clonal lineages using a wide range of IGHV genes (Fig. 2c). However, we observed skewing of the responding IGH repertoires away from the usually most frequently utilized IGHV genes such as IGHV3–23, towards IGHV3–30 and IGHV3–9 usage, with particular enrichment of IGHV3–9 in IgG-expressing B cells. The selection of particular IGHVs in response to an antigen has been observed in other antiviral responses, such as the preference for IGHV1–69 in response to some influenza virus antigens^9^. Highly utilized IGHV genes display SHM patterns consistent with the observed frequencies of unmutated or low mutation clonal lineages among switched isotype compartments (Fig. 2a), with low median IgG1 SHM that ranges from 2.2–5.4%, and higher median IgA1 SHM that ranges from 6.2–8.6% (Fig. 2d).

## Expanded B Cell Clones Correlate With Seroconversion And Show Distinctive Igh Features

**Figure 3:**
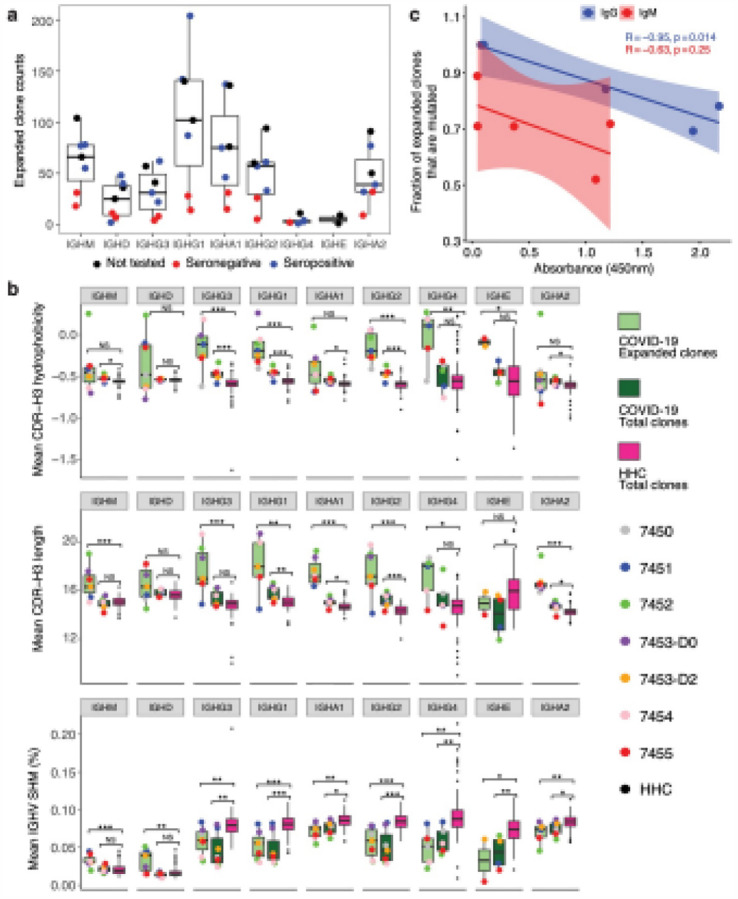
Expanded B cell clones in COVID-19 patients show selection for hydrophobic and longer CDR-H3s with less mutation. **a**, Expanded clone counts in COVID-19 patients. Each point represents the number of expanded clones (y-axis) detected in a patient sample that contain the specified isotype (x-axis). Point colors indicate serology not tested (black), seronegative (red), and seropositive (blue). **b**, Mean CDR-H3 hydrophobicity (top panel), mean CDR-H3 length (middle panel), and mean IGHV SHM (bottom panel) for COVID-19 patient expanded clones (light green) or total clones (dark green), or for total healthy human control (HHC) clones (pink, only outlier points are displayed for this group). Each point on the plot represents the average of expanded clones in a patient sample that express the indicated isotype (x-axis). Points are jittered on the x-axis to decrease over-plotting of samples with the same value (y-axis). p-values were calculated by two-sided Wilcoxon-Mann-Whitney tests: ***p-value < 0.001; **p-value < 0.01; *p-value ≤ 0.05; NS: p-value ≥ 0.05. c, Correlation between the serum anti-SARS-CoV-2-RBD levels measured by ELISA (x-axis) and the fraction of expanded clones with SHM ≥ 1 % (y-axis). Clones expressing IgG were correlated with levels of anti-SARS-CoV-2-RBD-IgG antibodies; clones expressing IgM were correlated with levels of anti-SARS-CoV-2-RBD-IgM antibodies. Each point represents a patient sample. Shaded regions indicate 95% confidence intervals. R2 and p were measured by Pearson’s correlation. Only individuals for whom serology was performed are shown (7450, 7451, 7452, 7453, and 7454).

To further explore antigen-driven B cell responses in the patient cohort, we identified expanded clonal lineages based on their presence in two or more replicate genomic DNA (gDNA) libraries generated from separate template aliquots isolated from patient peripheral blood mononuclear cells (PBMCs). This method avoids distortions of sequence counts in cDNA libraries caused by high mRNA expression from some cells^11^ (Fig. 3a and Extended Data Fig. 3). IGHV genes that were significantly enriched in expanded clones were determined based on the odds ratio (OR) of IGHV gene usage in expanded clones versus non-expanded clones included IGHV6–1 (OR = 2.3, p-value = 0.0004), IGHV3–33 (OR = 1.30, p-value = 0.005) and IGHV4–61 (OR = 1.44, p-value = 0.007). CDR-H3 regions from total clones and expanded-only clones in COVID-19 patient IGH repertoires display clear differences in physicochemical properties compared to total HHC CDR-H3 regions (Fig. 3b and Extended Data Fig. 4). SHM values for class-switched expanded clones are significantly lower in COVID-19 patients compared to healthy controls in sequences expressing all IgG and IgA subclasses (p-value < 0.01 for IgG3, IgG4, IgA1, and IgA2; p-value < 0.001 for IgG1 and IgG2), and significantly higher in IgM (p-value < 0.001) and IgD (p-value < 0.01). There is a strong correlation between the fraction of serum anti-SARS-CoV-2 receptor binding domain (RBD) IgG levels and the fraction of IgG-expressing clonally-expanded lineages in patient (R = 0.95, p-value = 0.014) (Fig 3c).

## Convergent Antibody Rearrangements Are Elicited In Covid-19 Patients

Although antigen-driven antibody responses are diverse between individuals, we and others have previously identified patterns of highly similar, “convergent” antibodies shared by individuals in response to Ebola virus^8^, and different convergent antibodies stimulated by Dengue virus^12^.

**Figure 4:**
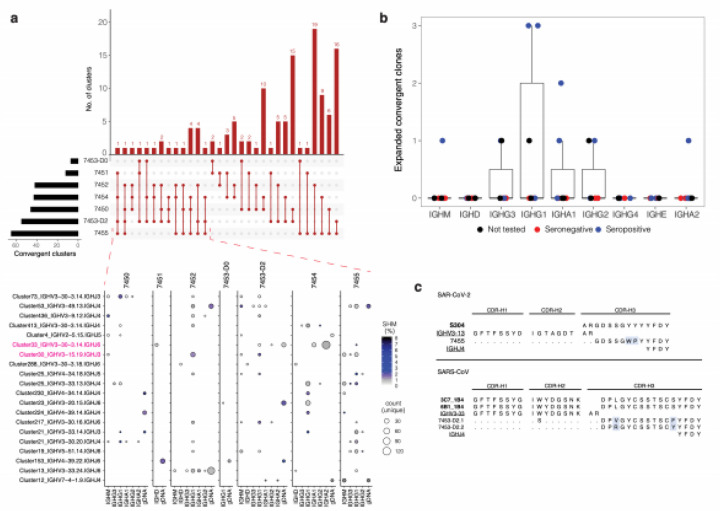
Convergent IGH sequences are found between COVID-19 patients. **a**, The distribution of convergent clusters among patient samples (top panel). The sample distribution is indicated by the lines and dots with the number of clusters sharing that sample distribution indicated by the vertical histogram bars. The total number of convergent clusters identified in each sample is indicated in the histogram to the left of the plot. (bottom panel) Lineages belonging to convergent clusters (y-axis) shared across three or four patients (columns, four-patient clusters are highlighted in pink) are plotted by the expressed isotype (x-axis). Fill color indicates the average SHM and point size shows the number of unique reads. **b**, Expanded convergent clone counts in COVID-19 patients. Each point represents the number of expanded convergent clones (y-axis) detected in a patient sample that express the specified isotype (x-axis). Point colors indicate serology not tested (black), seronegative (red), and seropositive (blue). **c**, Sequence alignment of CDR-H1, CDR-H2, and CDR-H3 amino acid residues of anti-SARS-CoV convergent IGH (rows). Sequences were aligned against a SARS-CoV and SARS-CoV-2 cross-reactive IGH (top) or SARS-CoV-specific IGH (bottom) sequences (labels in bold). Light blue highlighting indicates sequence differences. Dots indicate homology to the germline sequence (labels are underlined).

These convergent antibodies usually make up a small proportion of the total virus-specific B cell response in each individual. To identify putative SARS-CoV-2-specific antibody signatures, we first grouped heavy chain sequences together that utilized the same IGHV and IGHJ gene family, and CDR-H3 regions with the same length. We then clustered these groups by single-linkage with 85% identity in the CDR-H3 amino acid sequence and identified sequences that were found in at least two COVID-19 patients but were absent from the 114 HHC repertoires. We identified 124 such convergent clusters involving clonal lineages from all six patients. The number of convergent clusters that each patient contributed to varied from 12 clusters in patient 7451 to 65 clusters in patient 7455. 106 clusters were shared pairwise between two patients, 16 clusters spanned three patients, and two clusters spanned four patients (Fig. 4a). Clusters that spanned four subjects included a cluster of lineages using IGHV3-30-3 and IGHJ6 with a 14 amino-acid CDR-H3, which was expanded across different isotypes in 7453-D2 and 7454 with SHM frequencies generally below 2% and was detected as IgD in 7451 and IgG1 in 7455. We further detected one to three convergent clones in samples 7450, 7452, and 7453-D2 that were expanded as per the gDNA replicate analysis. Samples with expanded clones by this definition expressed IgM, IgG1–3, IgA1, and IgA2 (Fig. 4b). We next sought to identify SARS-CoV-2-specific sequences homologous to known sequences that recognize SARS-CoV-2 or the related betacoronavirus, SARS-CoV. Sequences convergent to known SARS-CoV-specific antibody IGH sequences^13^ were identified in one COVID-19 patient (7453-D2) but were not detected in the HHC samples (Fig. 4c). We also identified one IGH (IGHV3–13, IGHJ4, and CDR-H3 length of 14), recently reported to cross-react to SARS-CoV and SARS-CoV-2^14^, which clustered with IGH sequences identified in patient 7455 (Fig. 4c).

Our results indicate that the IGH repertoires of patients with diagnostically confirmed SARS-CoV-2 RNA and COVID-19 infection are rapidly remodeled in response to this novel virus. A burst of expanded clones with low SHM, shifts in the utilization of IGHV genes and subclasses, and longer and more hydrophobic CDR-H3 regions dominate the molecular changes observed in IGH. We observe convergent antibody responses shared with those seen in patients infected with SARS-CoV, highlighting common modes of human antibody response shared between SARS-CoV-2 and other spillover CoVs. Efforts to detect individuals who have been exposed to SARS-CoV-2 and have recovered after mild or asymptomatic infection have revealed delayed serum antibody responses and low levels of specific antibodies in some individuals^15^. Convergent SARS-CoV-2-specific antibody sequences in the memory B cell pools of recovered individuals could offer an independent kind of evidence of prior exposure and potential for more rapid secondary responses upon re-exposure to the virus. Longitudinal tracking of IGH repertoires in larger patient cohorts, correlation with clinical outcomes, and further investigation into the binding properties and functional activity of convergent responding clones, are required to better understand the human antibody responses to this novel human SARS-CoV-2.

## Data Availability

All data is available in the main text or the extended materials. The IGH repertoire data for this study have been deposited to SRA with accession number SUB7246339.

## Methods

### Study Design

Patients admitted to Stanford Hospital with signs and symptoms of COVID-19 and confirmed SARS-CoV infection by RT-qPCR of nasopharyngeal swabs were recruited. Venipuncture blood samples were collected in K2EDTA- or sodium heparin-coated vacutainers for peripheral blood mononuclear cell (PBMC) isolation or serology on plasma, respectively. Recruitment of COVID-19 patients, documentation of informed consent, collections of blood samples, and experimental measurements were carried out with Institutional Review Board approval (IRB- 55689).

### Healthy adult controls (HHCs)

The data set containing control immunoglobulin receptor repertoires has been described previously7. In summary, healthy adults with no signs or symptoms of acute illness or disease were recruited as volunteer blood donors at the Stanford Blood Center. Pathogen diagnostics were performed for CMV, HIV, HCV, HBV, West Nile virus, HTLV, TPPA (Syphilis), and *T. cruzi*. Volunteer age range was 17–87 with median and mean of 52 and 49, respectively.

### Molecular and serological testing on COVID-19 patients

SARS-CoV-2 infection in patients was confirmed by reverse-transcription polymerase chain reaction testing of nasopharyngeal swab specimens, using the protocols described in^16^,^17^. Plasma antibody testing for IgG and IgM specific for SARS-CoV-2 spike protein receptor binding domain (RBD) was carried out with an enzyme-linked immunosorbent assay based on the protocol and antigen protein production described in^18^.

### HTS of immunoglobulin heavy chain (IGH) libraries prepared from genomic DNA and cDNA

The AllPrep DNA/RNA kit (Qiagen) was used to extract genomic DNA (gDNA) and total RNA from PBMCs. For each blood sample, six independent gDNA library PCRs were set up using 100 ng template/library (25ng/library for 7453-D0). Multiplexed primers to *IGHJ* and the FR1 or FR2 framework regions (3 FR1 and 3 FR2 libraries), per the BIOMED-2 design were used^19^ with additional sequence representing the first part of the Illumina linkers. In addition, for each sample, total RNA was reverse-transcribed to cDNA using Superscript III RT (Invitrogen) with random hexamer primers (Promega). Total RNA yield varied between patients and between 6 ng-100 ng was used for each of the isotype PCRs using IGHV FR1 primers based on the BIOMED-2 design^19^ and isotype specific primers located in the first exon of the constant region for each isotype category (IgM, IgD, IgE, IgA, IgG). Primers contain additional sequence representing the first part of the Illumina linkers. The different isotypes were amplified in separate reaction tubes. Eight-nucleotide barcode sequences were included in the primers to indicate sample (isotype and gDNA libraries) and replicate identity (gDNA libraries). Four randomized bases were included upstream of the barcodes on the *IGHJ* primer (gDNA libraries) and constant region primer (isotype libraries) for Illumina clustering. PCR was carried out with AmpliTaq Gold (Applied Biosystems) following the manufacturer’s instructions, and used a program of: 95°C 7 min; 35 cycles of 94°C 30 sec, 58°C 45 sec, 72°C 60 sec; and final extension at 72°C for 10 min. A second round of PCR using Qiagen’s Multiplex PCR Kit was performed to complete the Illumina sequencing adapters at the 5’ and 3’ ends of amplicons; cycling conditions were: 95°C 15 min; 12 cycles of 95°C 30 sec, 60°C 45 sec, 72°C 60 sec; and final extension at 72°C for 10 min. Products were subsequently pooled, gel purified (Qiagen), and quantified with the Qubit fluorometer (Invitrogen). Samples were sequenced on the Illumina MiSeq (PE300) using 600 cycle kits.

### Sequence quality assessment, filtering, and analysis

Paired-end reads were merged using FLASH^20^, demultiplexed (100% barcode match), and primer trimmed. The V, D, and J gene segments and V-D (N1), and D-J (N2) junctions were identified using the IgBLAST alignment program^21^. Quality filtering of sequences included keeping only productive reads with a CDR-H3 region, and minimum V-gene alignment score of 200. For cDNA-templated IGH reads, isotypes and subclasses were called by exact matching to the constant region gene sequence upstream from the primer. Clonal identities were inferred using single-linkage clustering and the following definition: same IGHV and IGHJ usage (disregarding allele call), equal CDR-H3 length, and minimum 90% CDR-H3 nucleotide identity. A total of 518,403 clones (per sample, mean number of clones: 74,058; median number of clones: 9,030 for each isotype) were identified. A total of 6,158,222 IGH sequences amplified from cDNA were analyzed for the COVID-19 subjects (mean: 879,746 per individual; median: 910,437) and 68,831,446 sequences from healthy adult controls (mean: 603,785 per individual; median: 637,269). Each COVID-19 patients had on average 280,307 in-frame gDNA sequences and each adult control had an average of 8,402 in-frame gDNA sequences.

For each clone, the median somatic mutation frequency of reads was calculated. Mean mutation frequencies for all clonal lineages from a subject for each isotype were calculated from the median mutation frequency within each clone, and so represent the mean of the median values. Clones with <1 % mutation were defined as unmutated and clones with ≥ 1 % were defined as being mutated. Subclass fractions were determined for each subject by dividing the number of clones for a given subclass by the total number of clones for that isotype category. Expanded clones were defined as a clone found in one subject which is present in two or more of the gDNA replicate libraries. Clonal expansion in the isotype data was inferred from the gDNA data. Analyses were conducted in R^22^ using base packages for statistical analysis and the ggplot2 package for graphics^23^.

To determine convergent rearranged IGH among patients with SARS-CoV-2 infection, we clustered heavy-chain sequences annotated with the same IGHV and IGHJ segment (not considering alleles) and the same CDR-H3 length were clustered based on 85% CDR-H3 amino acid sequence similarity using cdhit^24^. To exclude IGH that are generally shared between humans and to enrich the SARS-CoV-2-specific IGH that are likely shared among the patients, clusters were selected as informative if (1) they contained at least five IGH sequences from each COVID-19 patient and were present in at least two subjects; (2) no IGH sequences from HHC samples (collected prior to the 2019 SARS-CoV-2 outbreak) were identified in the same convergent cluster. The same selection criteria were used to determine the convergent clusters between the COVID-19 samples and previously reported IGH sequences specific to SARS-CoV-1 and SARS-CoV-2.

## Supplementary Material

Supplement
