# Supplementary material for: B cell clonal expansion and convergent antibody responses to SARS-CoV-2
Source: Res Sq. 2020 May 6:rs.3.rs-27220. Preprint. [Version 1] doi: 10.21203/rs.3.rs-27220/v1 (PMC7336706; doi:10.21203/rs.3.rs-27220/v1)
Supplement: Supplement [file extendeddata.pdf]

Extended Data figures

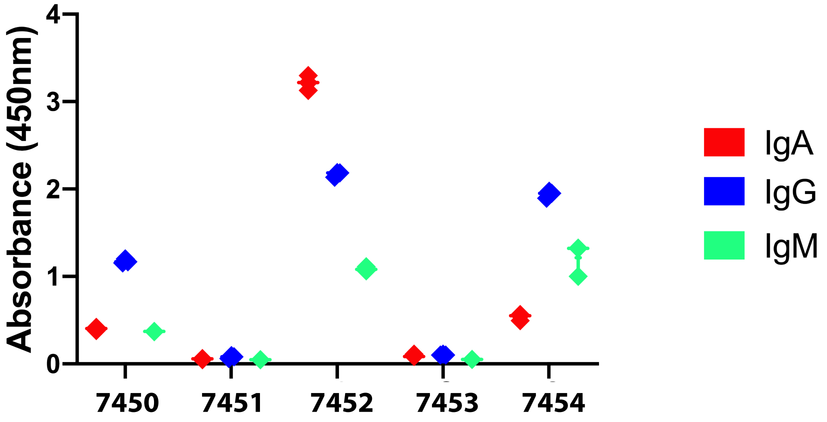

	IGHA			IGHG			IGHM		
**7450**	0.417	0.404	0.383	1.202	1.168	1.156	0.372	0.373	0.367
**7451**	0.06	0.061	0.049	0.061	0.082	0.096	0.049	0.047	0.047
**7452**	3.3	3.219	3.13	2.187	2.136	2.186	1.112	1.066	1.083
**7453-D0**	0.084	0.11	0.086	0.111	0.103	0.101	0.05	0.05	0.05
**7454**	0.553	0.566	0.496	1.951	1.896	1.976	1.324	1	1.323

Extended Data Fig. 1. Anti-SARS-CoV-2 RBD serology results. Plasma antibody levels of IgA, IgG, and IgM specific for SARS-CoV-2 RBD domain were measured by ELISA and reported as optical density values at 450nm. All values were measured in triplicate. Positive results were those with OD_{450} greater than a threshold of 0.3, based on the average plus three standard deviations of results obtained from assaying historical negative control blood donor specimens collected in 2018.

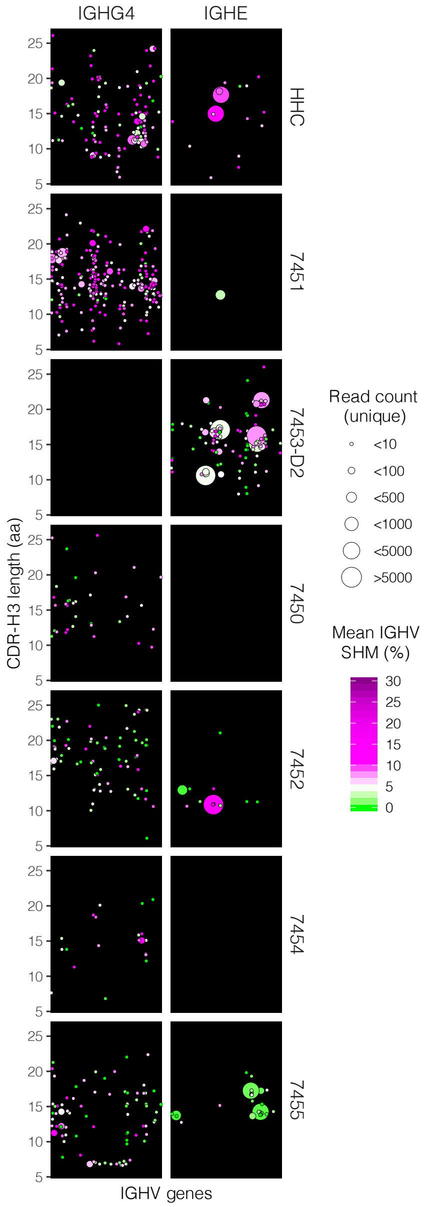

Extended Data Fig. 2. IGHG4 and IGHE IGH repertoire overview for COVID-19 patients. Each point in the plot represents a single B cell lineage and each position denotes the clone's isotype (column), subject (row), IGHV gene (x-axis), and CDR-H3 length (y-axis). The point color indicates the mean SHM frequency for each clone and the size indicates the number of unique reads grouped into the clone. Points are jittered to decrease over-plotting of clones with same IGHV gene and CDR-H3 length.

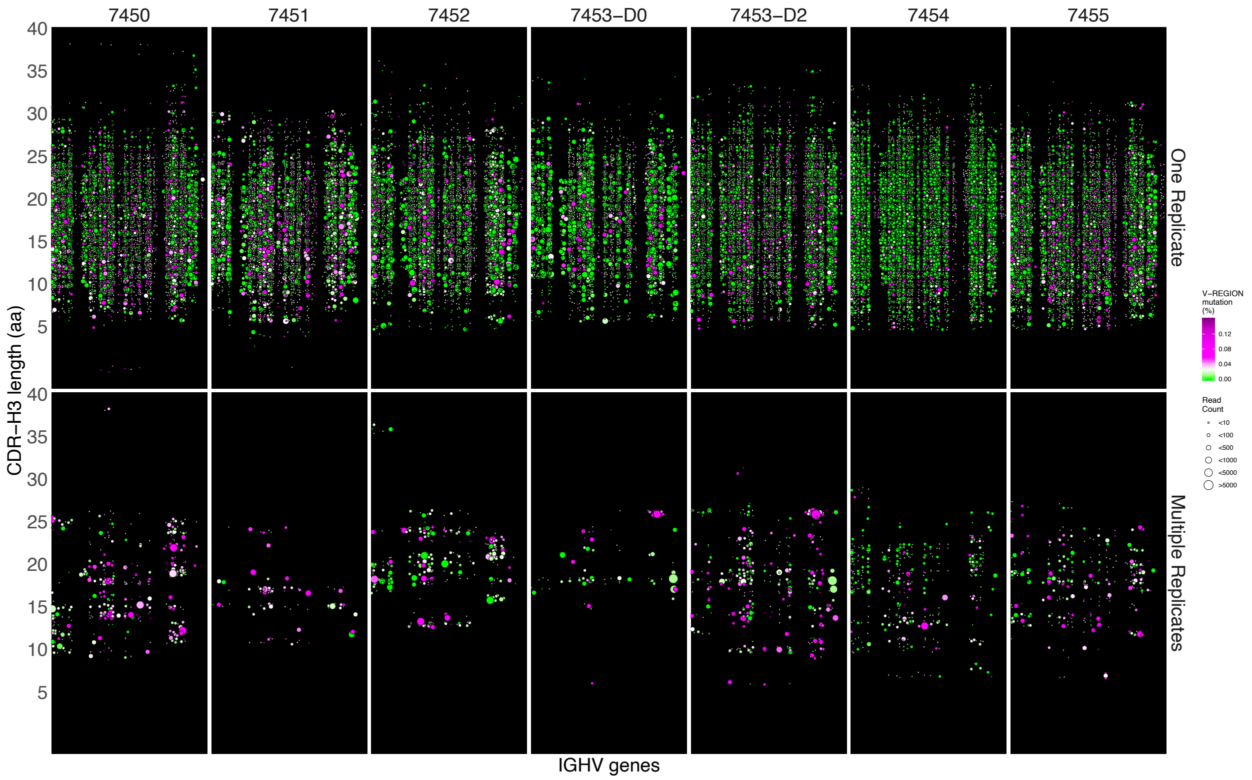

Extended Data Fig. 3. IGH repertoire for expanded clones in COVID-19 patients. Each point in the plot represents a single B cell lineage and each position denotes the clone IGHV gene (x-axis), CDR-H3 length (y-axis), the patient (column), and the number of gDNA replicates in which the clone was present (row). Expanded clones are those found in multiple replicates (bottom panel); non-expanded clones are found in a single replicate (top panel). The point color indicates the mean SHM frequency for each clone and the size indicates the number of unique reads grouped into the clone. Points are jittered to decrease over-plotting of clones with same IGHV gene and CDR-H3 length.

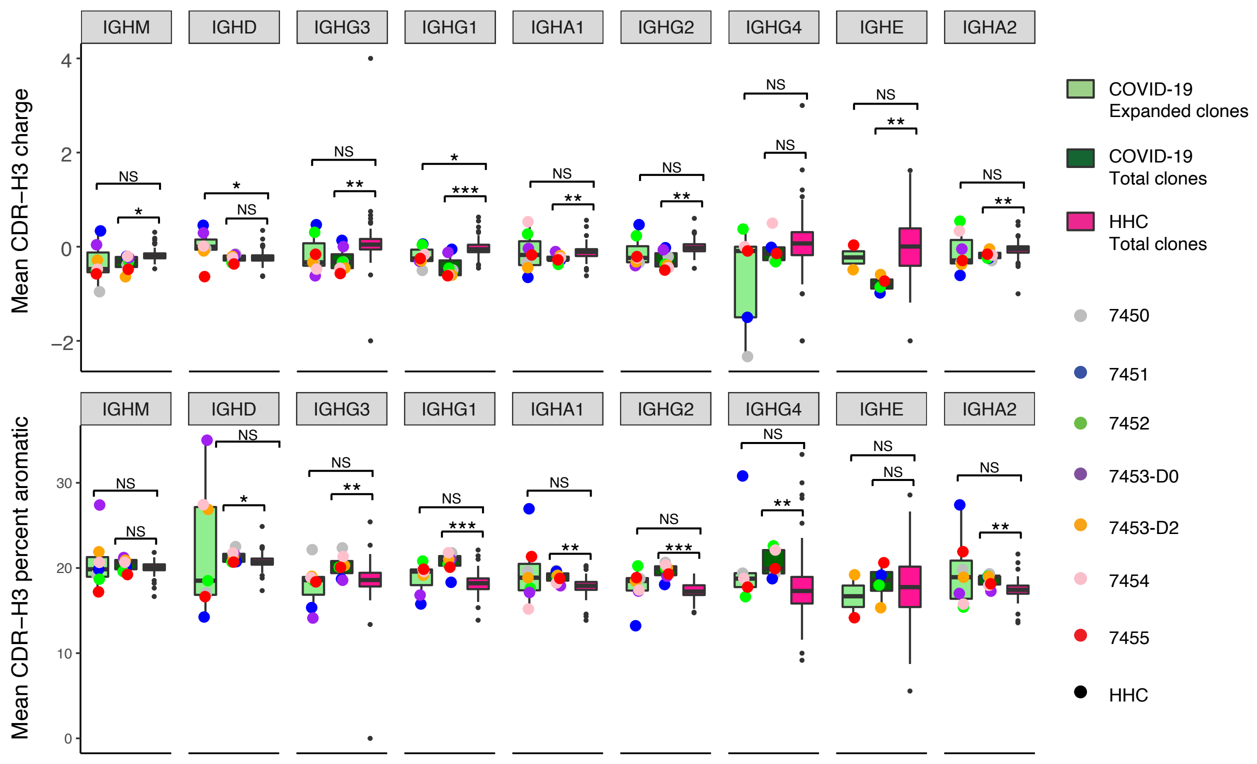

Extended Data Fig. 4. Additional CDR-H3 features of expanded clones. Mean charge (top panel) and percent aromaticity (bottom panel) for CDR-H3 amino acid residues in expanded clones (light green) and total clones from COVID-19 patients (dark green), and total clones from healthy human controls (pink). Each point on the plot represents a patient sample. Points are jittered on the x axis to decrease over-plotting of samples with the same CDR-H3 feature score (y-axis).
